# Supplementary material for: Investigating the influence of physiologically relevant hydrostatic pressure on CHO cell batch culture
Source: Sci Rep. 2021 Jan 8;11:162. doi: 10.1038/s41598-020-80576-8 (PMC7794228; doi:10.1038/s41598-020-80576-8)
Supplement: Supplementary file 1 — Supplementary Information 1. [file 41598_2020_80576_MOESM1_ESM.zip › S1/IgG/Jan17 standard curve/20ugml.pdf]

Data File C:\Users\P...s\ChemStation\1\Data\TK_CHO_IgG 2019-01-17 13-50-44\002-2-20ug_ml.D
Sample Name: 20ug/ml

===

Acq. Operator : SYSTEM Seq. Line : 2
Sample Operator : SYSTEM
Acq. Instrument : HPLC-0039 Location : 2
Injection Date : 1/17/2019 1:56:32 PM Inj : 1
Inj Volume : 100.000 µl
Acq. Method : C:\Users\Public\Documents\ChemStation\1\Data\TK_CHO_IgG 2019-01-17 13-50-44\CHO_IgG_LC.M
Last changed : 12/4/2018 1:38:41 PM by SYSTEM
Analysis Method : C:\Users\Public\Documents\ChemStation\1\Data\TK_CHO_IgG 2019-01-17 13-50-44\CHO_IgG_LC.M (Sequence Method)
Last changed : 1/17/2019 2:22:08 PM by SYSTEM
(modified after loading) (Current integration events modified)

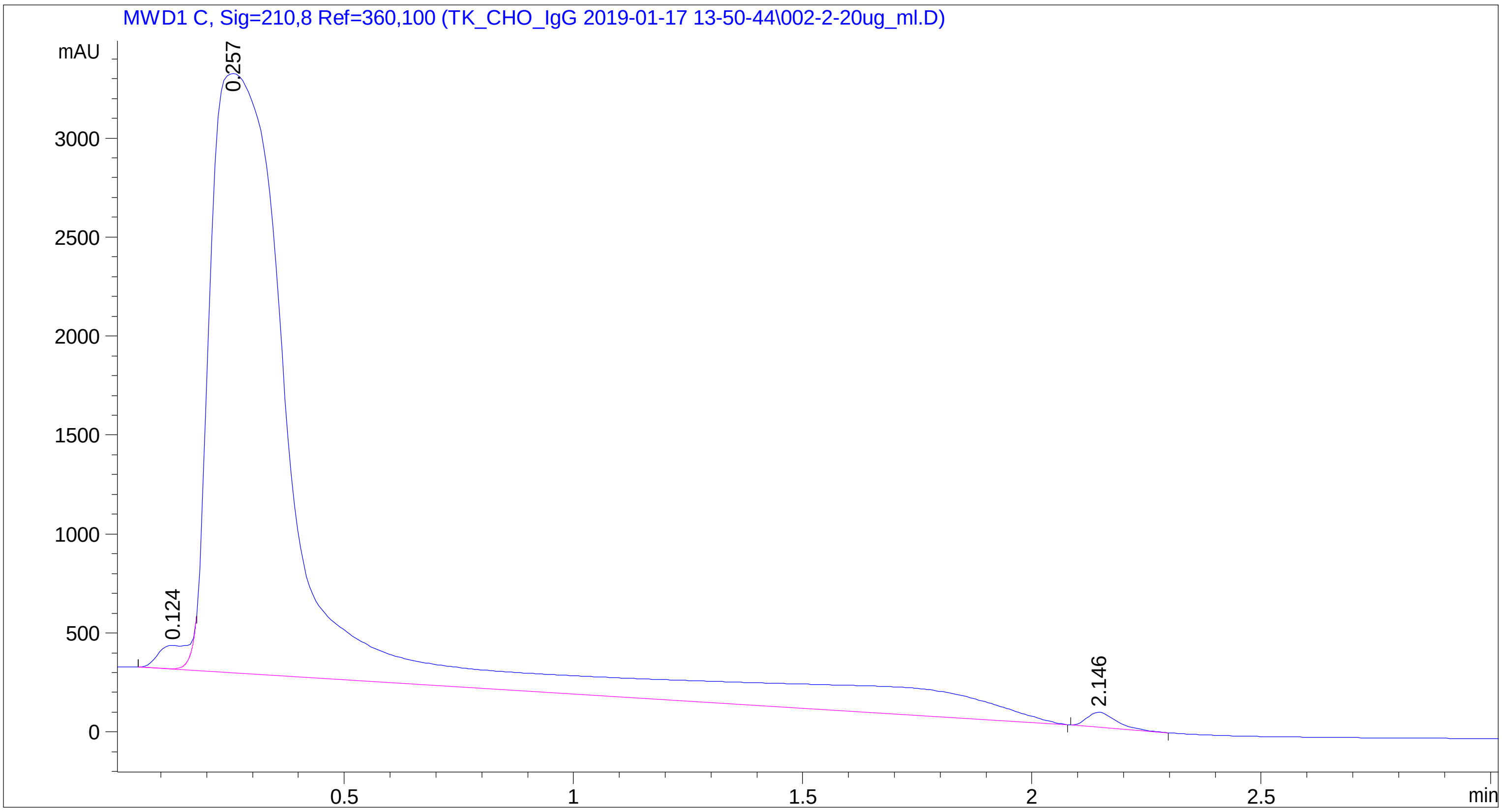

===

Area Percent Report

===

Sorted By : Signal
Multiplier : 1.0000
Dilution : 1.0000
Use Multiplier & Dilution Factor with ISTDs

Signal 1: MWD1 C, Sig=210,8 Ref=360,100

Peak #	RetTime [min]	Type	Width [min]	Area [mAU*s]	Height [mAU]	Area %
1	0.124	BV E	0.0600	501.00778	116.91795	1.1638
2	0.257	VB R	0.2108	4.22191e4	3026.24390	98.0753
3	2.146	BBA	0.0647	327.53839	75.34753	0.7609

Totals : 4.30476e4 3218.50938

===

*** End of Report ***
